# Microstructure, local dynamics, and flow behavior of colloidal suspensions with weak attractive interactions

**DOI:** 10.1038/srep33498

**Published:** 2016-09-22

**Authors:** Clara Weis, Claude Oelschlaeger, Dick Dijkstra, Meik Ranft, Norbert Willenbacher

**Affiliations:** 1 Karlsruhe Institute for Technology (KIT), Institute for Mechanical Process Engineering and Mechanics, Applied Mechanics, Karlsruhe, 76131, Germany; 2 Covestro Germany, Leverkusen, 51365, Germany; 3 BASF SE, Ludwigshafen, 67056, Germany

## Abstract

We present a comprehensive micro- and macrorheological study of the effect of weak depletion attraction (Ψ_dep_ ≈ 1–10 k_B_T) on dense colloidal suspensions stabilized by short-range repulsive interactions. We used aqueous polymer dispersions as model system and demonstrated the unique capabilities of multiple particle tracking (MPT) to disclose structural changes in such technically important systems exhibiting many characteristic features of hard sphere systems. Below the hard sphere freezing point ϕ_c_, viscosity increases monotonically with increasing Ψ_dep_ due to the transition from a fluid to a fluid/crystalline and finally to a gel state. Above ϕ_c_, increasing attraction strength first results in a viscosity reduction corresponding to the formation of large, permeable crystals and then in a viscosity increase when a network of dense, small crystals forms. The fraction of the fluid and crystal phase, particle concentration in each phase as well as the modulus of the micro-crystals are obtained, the latter decreases with Ψ_dep_. Above the colloidal glass transition strong heterogeneities and different local particle mobility in the repulsive and attractive arrested states are found. Particles are trapped in the cage of neighboring particles rather than in an attractive potential well. The intermediate ergodic state exhibits uniform tracer diffusivity.

Understanding phase behavior, structure, and dynamics of colloidal dispersions is of utmost importance for two complementing reasons. First, they serve as model systems for studying fundamental physical phenomena like crystallization, gelation, and glass formation at conveniently accessible time and length scales^1^,^2^,^3^. Second, they are widely used in the fabrication of nanostructured materials in emerging innovative fields of application, e.g., photonic crystals, and in well-established commodity coatings and adhesives, often as precursors enabling the processing of materials after drying and sintering for final use in their solid state. A key technologic challenge is to control their flow properties to meet the many requirements of processing and application. Numerous experimental and theoretical investigations as well as numerical simulations have addressed the above-mentioned phenomena, and so-called hard sphere model systems characterized by just one physical parameter, namely the volume fraction ϕ, are used to study condensed matter physics^4^,^5^,^6^. It must be kept in mind, however, that the hydrodynamic interactions among suspended colloids mediated by the surrounding solvent are different than the interactions in molecular fluids. They affect diffusivity^7^, high frequency rheology^8^, and even crystallization^9^, thus somewhat limiting the ability to transfer the results found for colloidal suspensions to molecular fluids. Nevertheless, hard sphere colloidal model suspensions are of invaluable importance for understanding colloidal suspensions in general. In particular, phase behavior and flow properties of suspensions with short-range repulsive particle interactions can be qualitatively described using the effective volume fraction ϕ taking into account repulsive thermodynamic forces (hard sphere mapping)^10^.

In addition to macroscopic rheological experiments and scattering techniques, confocal scanning microscopy is a powerful tool for studying the structure and dynamics of colloidal hard sphere systems. Dynamic heterogeneities and structural relaxation phenomena near the colloidal glass transition^11^,^12^, have been investigated as well as the microscopic structure of shear thinning and shear thickening suspensions^13^. In these experiments, the thermal motion of several thousand particles is tracked with high spatial and temporal resolution^14^. The particle diameter is typically between 1 and 5 μm and polymer particles are suspended in appropriate organic solvent mixtures to provide refractive index matching, not only because of technical imaging issues, but also to mimic hard sphere properties as closely as possible. In some studies, the suspensions are density matched to avoid sedimentation and, particularly, gravitational effects on crystallization^15^. Technically relevant polymer dispersions, however, typically contain particles that are an order of magnitude smaller; not index-matched to the solvent; solved in water; not likely to form a sediment; and are stabilized via short-range steric, electrosteric, and electrostatic repulsion to provide a shelf life of at least several months. Such suspensions are utilized as emerging innovative materials^16^,^17^, but, more importantly, today they are used in numerous kinds of coatings or adhesive products and annual global production is on the order of 10 million tons. Accordingly, new insight into their structure and processability has a potentially huge impact on a wide range of fields. Here we present comprehensive microrheological studies on such turbid aqueous colloidal polymer dispersions covering a broad concentration range from the dilute to the glassy state. Our focus is on the effect of weak attractive interactions induced by non-adsorbing polymers dissolved in the aqueous phase. We used multi particle tracking (MPT) microrheology, i.e., we simultaneously tracked the Brownian motion of several hundred fluorescent tracer particles added to a colloidal suspension. This technique was originally developed to study cell biology systems^18^,^19^, but was later applied to study heterogeneity, e.g., in clay suspensions^20^,^21^, polymeric thickener solutions^22^,^23^, and agarose^24^ and food gels^25^. Here, we applied it to highly turbid colloidal suspensions for the first time. Particle trajectories can be tracked within a focal plane ∼30 μm deep within these samples, which corresponds to more than 100 particle diameters, i.e., artifacts due to the sample surface can be excluded. One can calculate the linear viscoelastic modulus G* from the time dependence of the mean square displacement (MSD) of individual particles^26^, and for homogeneous fluids this agrees well with the macroscopic modulus obtained from bulk mechanical rheometry. Here we calculate the viscosity η_MPT_ from MSDs of freely diffusing tracers in a viscous fluid


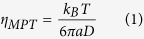


where k_B_ is the Boltzmann constant, T is the temperature, a is the particle radius, and D is the diffusion coefficient related to the MSD depending linearly on lag time Δr^2^(τ) = 4Dτ. Tracers trapped in crystalline or glassy domains exhibit a time-independent MSD directly related to the shear modulus of these regions.


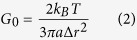


To characterize sample heterogeneities, we analyzed the distribution and slopes of the MSDs. We further calculated the van Hove correlation functions, i.e., the probability distribution of particle displacement for an ensemble of N tracked particles as:


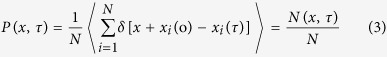


Where N(x, τ) is the number of particles found at positions between x and x + dx along the x-coordinate. P(x, τ) is Gaussian if all tracer particles are exposed to a similar environment. Deviations from this functional form reflect the presence of spatial heterogeneities and can be characterized by the non-Gaussian parameter α.


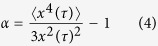


in which x^4^ and x^2^ are the fourth and second moments of P(x, τ). For a Gaussian distribution α = 0, experiments even on homogeneous systems usually yield α values slightly larger than zero but sample heterogeneities result in much larger values α ≫ 0. Finally, we used Voronoi triangulation^27^ and image overlay techniques to visualize the length scale of the spatial heterogeneities. This allowed us to study the non-trivial relationship between macroscopic flow behavior and sample composition (crystal fraction, size and densities) that comes with the broadening of the fluid/crystalline co-existence regime due to the introduction of weak attractive particle interactions^28^. We also studied changes in heterogeneity related to the so-called re-entry phenomenon, i.e., the fluidization of colloidal systems at particle loadings beyond the hard sphere glass transition, ϕ > ϕ_g,HS_ = 0.58, induced by a weak depletion attraction^29^.

## Results

Here, we present and discuss the results obtained for different colloidal dispersions stabilized by short-range electrosteric repulsion. Details about the chemical composition and particle size distribution are provided in the Materials and Methods section. For comparison of the results from different systems among each other and with published data, we determined an effective volume fraction from the divergence of the zero shear viscosity and, for brevity, we use the term ϕ for this quantity in the following text.

### Fluid State

**Figure 1:**
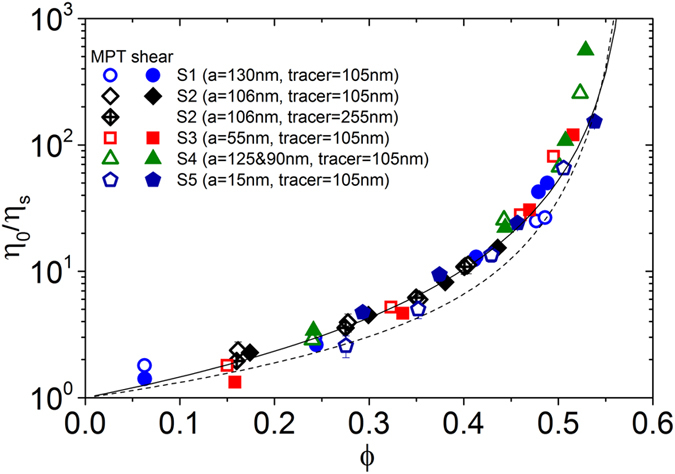
Variation of the relative viscosity, normalized by the solvent viscosity (η_s_ = 1 mPas), as a function of the volume fraction of various colloidal polymer dispersions with short range repulsive interactions as obtained from MPT (open symbols) and steady zero-shear measurements (closed symbols). The solid line represents the empirical Maron&Pierce model (Eq. 5) and the dashed line corresponds to the MCT based model given by Eqs 6 and 7. The chemical composition of each dispersion is summarized in Table 2.

We performed steady shear viscosity and MPT measurements of several colloidal dispersion systems in the fluid state at effective volume fractions of 0.05 < ϕ < 0.5. In all cases, the Brownian particle relaxation time τ_B_ = 6πηa^3^/k_B_T was less than 0.5 s and Δr^2^ ∼ τ, indicating free diffusion in a viscous fluid. Representative data are provided as supplementary information in Supplementary Fig. S1. The distribution of MSDs was narrow and the α-values were always close to zero, i.e., these suspensions behaved like homogeneous viscous fluids and we calculated η_MPT_ according to Eq. 1 from the ensemble-averaged MSD. In the steady shear experiments, all samples exhibited a constant Newtonian viscosity value at shear rates 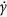 < 1s^−1^ as expected according to the short Brownian relaxation times. Corresponding macroscopic zero-shear viscosities η_0_ and the η_MPT_ data are plotted in Fig. 1 versus the volume fraction ϕ. All viscosity data are normalized to the solvent viscosity η_s_. Besides experimental data Fig. 1 displays two widely used models relating viscosity η_0_ and particle volume fraction ϕ. The empirical model proposed by Maron and Pierce^30^ is used here assuming that the viscosity diverges at the colloidal glass transition ϕ_g_:


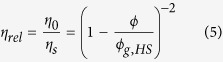


The second model equation derived from mode coupling theory (MCT)^31^ correctly predicts the dilute limit behavior of the viscosity to O(ϕ^2^) and its divergence at ϕ_g_:


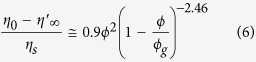


The high frequency viscosity η′_∞_ controlled by hydrodynamic interactions is calculated using the validated semi-empirical equation suggested by Lionberger and Russell^32^:


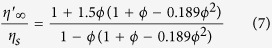


We found good overall agreement between η_MPT_ and η_0_ data for the large number of samples investigated and the concentration dependence of the normalized zero shear viscosity is fairly well described by both models introduced above. However, at intermediate particle volume fractions the empirical Maron&Pierce equation is closer to the experimental data than the MCT based model. Therefore, we will employ the former in the subsequent analysis of MPT data referring to suspensions in the fluid/crystalline co-existence regime. Supplementary Fig. S2 shows the scatter of the experimental data, but there is no systematic deviation from this empirical equation (Eq. 5) for the bulk or for the micro-viscosity. Our findings are different from previously reported results revealing the viscosity obtained from passive microrheology to agree very well with the macroscopic high frequency viscosity^33^. This is due to fact that this previous study used polymethylmethacrylate spheres with a τ_B_ = a^2^/D, which is orders of magnitude larger (a = 860 nm and 960 nm) than used in the present study based on particles with a = 130 nm and 106 nm, respectively.

Similar to Wilson *et al.*^33^, we did not obtain systematic deviations between micro-viscosity (i.e., long-time self-diffusivity) and macro-viscosity (here, zero-shear viscosity η_0_). This is intriguing because simulations as well as theoretical considerations suggest that the macro-viscosity of colloidal dispersions has a different dependence on particle volume fraction and should be systematically higher than the micro-viscosity for hard spheres, even if the volume effects are excluded, i.e., thermodynamic repulsion is considered^34^,^35^,^36^. This is presumably due not only to experimental uncertainties in measuring η_0_ and η_MPT_, but also to uncertainties in determining ϕ. Finally, it should be noted that the determination of η_MPT_ was robust with respect to the size of the probe particles; excellent agreement between corresponding data was found for the polyurethane dispersion S2 at different volume fractions when η_MPT_ was determined from the thermal motion of tracer particles with a radius of either 105 nm (similar to the dispersion particles) or 255 nm.

### Fluid-crystalline coexistence regime

The transition from the homogeneous fluid state to the regime in which fluid and crystalline domains with different particle concentrations coexist, ϕ_fluid_ and ϕ_solid_, respectively, shows up not only with a drastic increase in macroscopic low shear viscosity and strong shear-thinning (see Supplementary Fig. S1), but also in a pronounced drop of the mean MSD and its weak, sub-diffusive dependence on lag time τ. Beyond that, a broad distribution of absolute values and time-dependence of the observed MSDs is found because some tracers are trapped in the crystalline regions while others freely diffuse in the fluid regions. The characteristic microstructural length scale of the two-phase systems can be directly visualized based on a Voronoi triangulation of the video images using the center-of-mass coordinates of the tracer particles^23^. The difference in the logarithmic MSD slopes δ = d log(MSD)/d log(τ) can be used as a contrast and results in a black (δ ≤ 0.5, elastically trapped, crystalline) and white (δ ≥ 0.5, diffusive, fluid) contrast characterizing the dispersion microstructure (see Supplementary Fig. S1).

**Figure 2:**
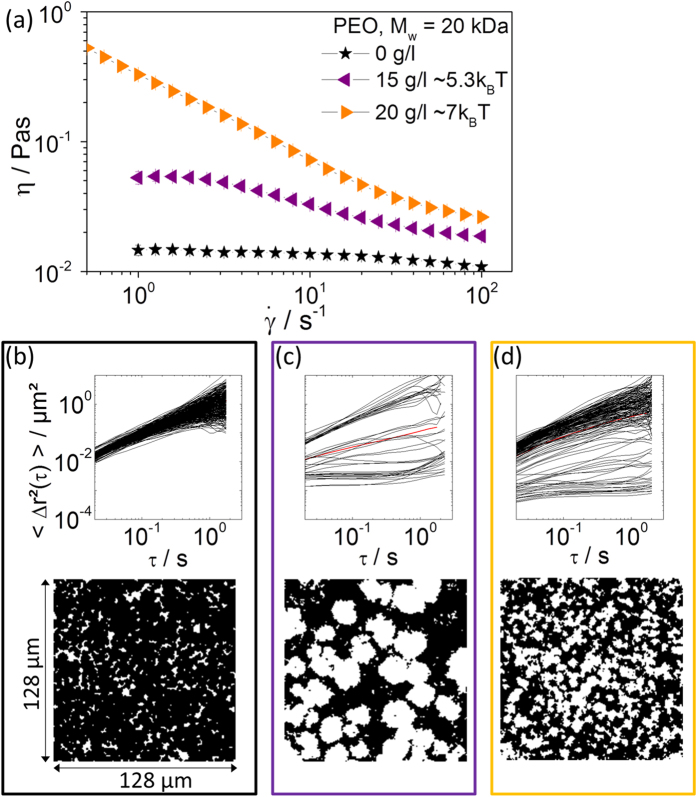
(**a**) Viscosity of the dispersion S1 (a = 130 nm, ϕ = 0.45) as a function of shear rate 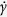 without added polymer (black stars), and with 15 g/l (Ψ_dep_ ≈ 5.3 k_B_T) (purple triangles) and 20 g/l (Ψ_dep_ ≈ 7 k_B_T) (yellow triangles) non-adsorbing polymer PEO (M_w_ = 20 kDa). MPT microrheology results obtained for the dispersion without added polymer **(b)**, and with 15 g/l **(c)** and 20 g/l PEO **(d)** with tracer particles of radius 105 nm. Frames are color coded according to the colors in (**a**). Upper row: mean square displacements (MSDs) of individual polystyrene microspheres as a function of lag time. Lower row: overlay of 1000 subsequent 128 × 128 μm images.

Here we focused on the effect of weak depletion attraction on the microstructure and macroscopic flow behavior of colloidal dispersions in the two-phase co-existence regime. Weak attractive interactions on the order of a few k_B_T among colloidal particles not only result in a broadening of the fluid/crystalline co-existence region, but also affect the size of the formed crystallites. Increasing the attraction strength further should result in gelation^37^. Figure 2 shows the steady shear viscosity as a function of shear rate, the time-dependence of the MSDs, and an overlay of 1000 subsequent images covering a time period Δτ = 10 s for a dispersion with ϕ = 0.45 and different concentrations of non-adsorbing polymer (poly(ethylene oxide) [PEO], M_w_ = 20 kg/mol). Characteristic data extracted from these experiments are summarized in Table 1.

Without the added polymer, the dispersion is in the fluid state with a low zero-shear viscosity and weak shear thinning behavior. The individual MSD traces lie in a narrow range, the non-Gaussian parameter α is close to zero, indicating that the sample is homogeneous, i.e., all tracer particles explore a similar environment. The slope of the MSD traces is δ ≈ 1, confirming free diffusion in a viscous medium and essentially the whole sample is explored by the diffusing tracers resulting in an almost uniformly black overlay image.

Introducing weak attraction (ϕ_polymer_ = 15 g/l, Ψ_dep_ = 5.3 k_B_T) results in an approximately four-fold increase in zero-shear viscosity and more pronounced shear thinning. Visual inspection of the samples reveals the opalescent characteristic of colloidal crystals. MPT reveals a broad variation in absolute values and the characteristic slopes δ of the MSD traces. The overlay image shows the size and shape of the crystalline regions. The flow behavior of the two-phase system can be simply modeled by treating the crystalline objects as spheres suspended in a Newtonian fluid comprising a diluted colloidal suspension. The zero shear viscosity is then given by Eq. 5. Inserting η_0_ = 53.4 ± 6 mPas from bulk rheometry and setting η_s_ = η_fluid_ = 3.8 ± 1.6 mPas as obtained from the MSDs with δ ≈ 1 results in a value 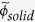 = 0.44 ± 0.04, which is in fair agreement with the crystalline volume fraction ϕ_solid_ = 0.51 ± 0.03 estimated from the white area in the overlay image (see Table 1).

The scenario changes drastically when the polymer concentration is further increased to ϕ_polymer_ = 20 g/l (Ψ_dep_ ≈ 7 k_B_T). The opalescence disappears and the steady shear viscosity further increases, shear thinning is more pronounced, and zero-shear viscosity is no longer accessible. This is characteristic of an attractive gel. MPT experiments again show a broad variety of MSDs with different absolute values and slopes. The microstructure of this gel state with its dense (white) and less dense (black) regions is seen in the overlay image, the characteristic length scale of this structure is 2.5 ± 0.7 μm, i.e., much finer than for the fluid/crystalline structure observed at a lower PEO concentration. We would like to emphasize here that the transition from the fluid/crystalline co-existence to the gel state occurs at higher reduced polymer concentrations than predicted by MCT^38^,^39^ and observed experimentally for suspensions of micrometer-sized hard sphere particles^40^. Moreover, we clearly observe a heterogeneous structure resembling a percolating network of dense clusters in a dilute suspension more than a uniform gel structure due to localization of individual particles as assumed in MCT. We assume that these discrepancies originate from the finite range of the repulsive interaction among the particles. This also shows up in the re-entry phenomenon at ϕ > ϕ_g,HS_ which will be discussed below. Colloidal systems with short range repulsion exhibit a much broader fluid regime than predicted by MCT for ideal hard spheres^41^ and Bartsch *et al.*^42^ have shown that the re-entry regime expands with decreasing steepness of the repulsive potential. Note, there was no indication of the existence of crystallites when the macro- and microrheological experiments were performed. Finally, we emphasize that at this particle loading, the viscosity increases monotonically with increasing polymer concentration.

**Figure 3:**
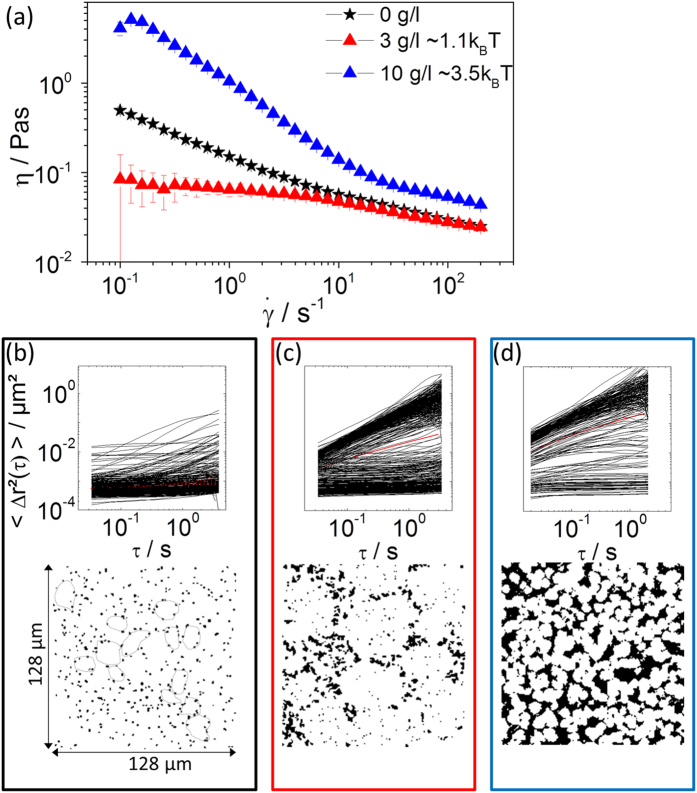
(**a**) Viscosity of the dispersion S1 (a = 130 nm, ϕ = 0.54) as a function of shear rate 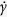 without added polymer (black stars), and with 3 g/l (Ψ_dep_ ≈ 1.1 k_B_T) (red triangles) and 10 g/l (Ψ_dep_ ≈ 3.5 k_B_T) (blue triangles) non-adsorbing polymer PEO (M_w_ = 20 kDa). MPT microrheology results obtained for the dispersion without added polymer **(b)**, and with 3 g/l **(c)** and 10 g/l PEO **(d)** with tracer particles of radius 0.105 nm. Frames are color coded according to the colors in (**a**). Upper row: mean square displacements (MSDs) of individual polystyrene microspheres as a function of lag time. Lower row: overlay of 1000 subsequent 128 × 128 μm images. For better illustration, dotted lines mark some crystals in (**b**).

The situation is different at ϕ values above the hard sphere freezing point, ϕ > 0.5. Here we investigated suspensions with different particle loadings and polymer concentrations (ϕ = 0.52–0.61, ϕ_polymer_ = 0–10 g/l), all samples exhibited opalescence and characteristic Bragg peaks could be observed in light diffraction experiments, i.e., all samples were in the fluid-crystalline co-existence regime. For example, Fig. 3(a) shows the viscosity curves for suspension S1 with ϕ = 0.54, but different amounts of added polymer. A substantial reduction in viscosity was observed for ϕ_polymer_ = 3 g/l whereas η drastically increased at ϕ_polymer_ = 10 g/l compared to the pure suspension. Similar results with a pronounced minimum in low shear viscosity as a function of polymer concentration were found for other particle loadings and were also reported previously for a similar colloidal suspension, but at different ϕ, ϕ_polymer_, and M_w_^43^.

The origin of this phenomenon is revealed by the MPT experiments and the corresponding data are shown in Fig. 3(b–d). Without added polymer, a broad variety of MSD traces with different absolute values and slopes is obtained and the overlay image clearly shows domains where the tracer particles hardly diffused, and, based on the opalescent appearance of the samples, we identified these domains as colloidal crystals. Image analysis yielded a mean crystallite radius of 8.2 ± 2.6 μm. When only 3 g/l of polymer was added, the tracer particle mobility changed remarkably. The freely diffusing tracers became more mobile and we observed two distinct populations of freely-diffusing and elastically-trapped particles. The corresponding overlay image reveals that the radius of the crystalline domains strongly increased to 15.3 ± 6.5 μm and these crystals were suspended in a fluid environment with increased tracer mobility, i.e., lower viscosity. This latter finding was expected based on the broadening of the co-existence region due to weak attractive depletion forces^44^, but the size of the crystals and the bulk viscosity were not clear a priori. We calculated η_fluid_ = 42 ± 7 mPas from the MSDs with δ ≈ 1 and estimated ϕ_solid_ = 0.73 ± 0.04 from the area of the white domains in the overlay image. This is close to the packing limit of spheres in an ordered fcc-structure and should thus result in a very high viscosity. The bulk viscosity η_0_, however, was only approximately twice the fluid viscosity η_fluid_ and we attributed this to the high permeability of the crystals with a particle density 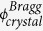 = 0.584 ± 0.007, as obtained from the diffraction experiments. When ϕ_polymer_ was increased to 10 g/l, the mobility of the freely-diffusing tracers further increased as expected because the broadening of the co-existence regime corresponded to a lower particle concentration in the fluid phase and a denser packing of the crystals, static light scattering provides 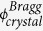 = 0.646 ± 0.009. Beyond that, the overlay images reveal that the size of the crystals was smaller than without the polymer. In this case, the flow behavior could again be modeled by treating the crystals as hard spheres suspended in a Newtonian liquid comprising a diluted dispersion. The volume fraction 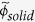 ≈ 0.56 ± 0.01 calculated by inserting η_0_ and η_fluid_ into Eq. 5 nicely agreed with the value ϕ_solid_ = 0.6 ± 0.03 directly obtained from the MPT (see Table 1).

Similar results regarding variation of bulk viscosity and crystal size with polymer concentration were obtained for ϕ = 0.52 and we concluded that the pronounced viscosity minimum observed at low polymer concentrations was directly related to the formation of large regular-shaped, but highly permeable, crystals.

**Figure 4:**
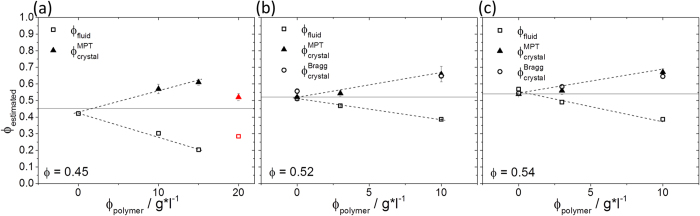
Estimated particle volume fraction in the fluid phase ϕ_fluid_ (open squares) and crystalline domain 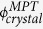 (filled triangles) as a function of polymer concentration for dispersion S1 for initial volume fractions: ϕ = 0.45 **(a)**, 0.52 **(b)**, and 0.54 **(c)**, as determined from the MPT measurements. In (**b**,**c**), 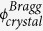 (open circles) represents the particle volume fraction in the crystalline domain determined from light diffraction experiments. The red data points in (**a**) correspond to the heterogeneous gel structure at high ϕ_polymer_.

MPT is also a valuable tool for exploring the phase diagram of colloidal suspensions. We calculated η_fluid_ from the MSDs of the freely-diffusing tracers and calculated the particle volume fraction of the fluid phase ϕ_fluid_ using Eq. 5 with η_fluid_ inserted as η_0_. The particle density in the crystal domains ϕ_crystal_ was obtained from light diffraction experiments, but it can also be deduced directly from the MPT data using the area fraction A_fluid_ pervaded by the tracer particles and, assuming that this quantity represents the fraction of the dispersion that is in the fluid state, we can set


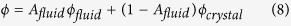


to calculate 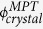. The corresponding results shown in Fig. 4 and Table 1 reveal good agreement between the ϕ_crystal_ values obtained from MPT and diffraction experiments. Furthermore, these data quantitatively demonstrate that the co-existence regime monotonically broadens with increasing amounts of polymer up to ϕ_polymer_ = 15 g/l. The gel state observed for ϕ = 0.45 and ϕ_polymer_ = 20 g/l is distinguished by a smaller variation in local particle concentration.

**Figure 5:**
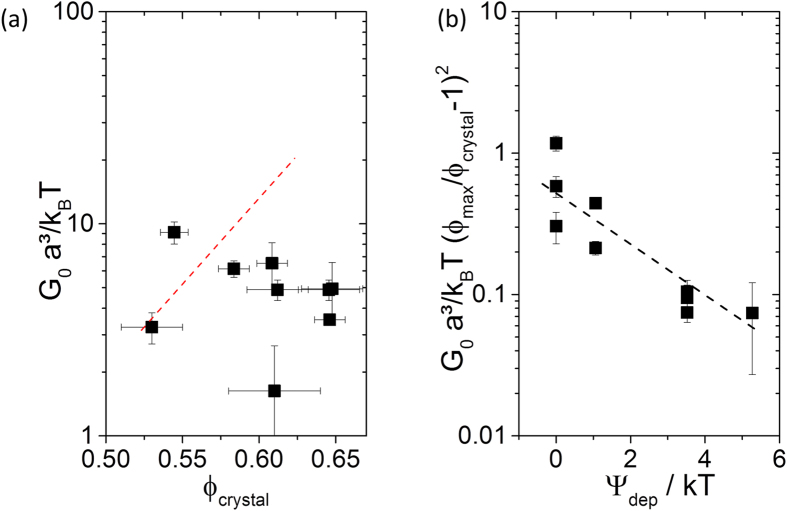
(**a**) Variation of the normalized plateau modulus G_0_ as a function of the estimated particle volume in the crystalline domain ϕ_crystal_ as determined from the MPT measurements. The red dotted line represents previous experiments^15^ and simulations for hard sphere systems^45^. **(b)** Variation of the reduced plateau modulus G_0_a^3^ = k_B_T(ϕ_max_/ϕ_crystal_ − 1)^2^ as a function of the depletion attraction potential Ψ_dep_, including particle volume fraction scaling as suggested by^45^. In this case, ϕ_max_ is set as the maximum volume fraction of a hcp-crystal (0.74). The dotted line serves as a guide to the eyes.

Finally, MPT can also be used to calculate the modulus of individual micron-sized crystals in highly turbid dispersions. Tracer particles incorporated into the colloidal crystals exhibited time-independent MSDs (≈0.05 a^2^) and modulus values obtained from Eq. 2 are shown in Fig. 5(a) for a particle density range of 0.52 < ϕ_solid_ < 0.65. Dimensionless data G_0_a^3^ = k_B_T are plotted in Fig. 5(a) to compare with previous experimental^15^ and simulation results^45^. The absolute values determined here agree very well with the older data. Despite the large experimental uncertainty, however, there was no indication of a monotonic increase of G_0_ with ϕ_crystal_ as observed earlier; here the modulus of the crystals was essentially independent of particle density. Crystallization in hard sphere dispersions is an entropy-driven phenomenon, the ordered state provides a higher configurational entropy for the individual particles. This contribution to the free energy of the system is changed due to the attractive interactions that are especially present at high ϕ_crystal_. Figure 5(b) directly shows that the reduced modulus G_0_a^3^ = k_B_T(ϕ_max_/ϕ_crystal_ − 1)^2^ decreases monotonically with increasing attraction strength. The modulus data were normalized here with respect to particle concentration, as suggested in^45^, ϕ_max_ = 0.74 denotes the maximum packing fraction of a hexagonal closed packed (hcp) crystal.

### Glassy state

**Figure 6:**
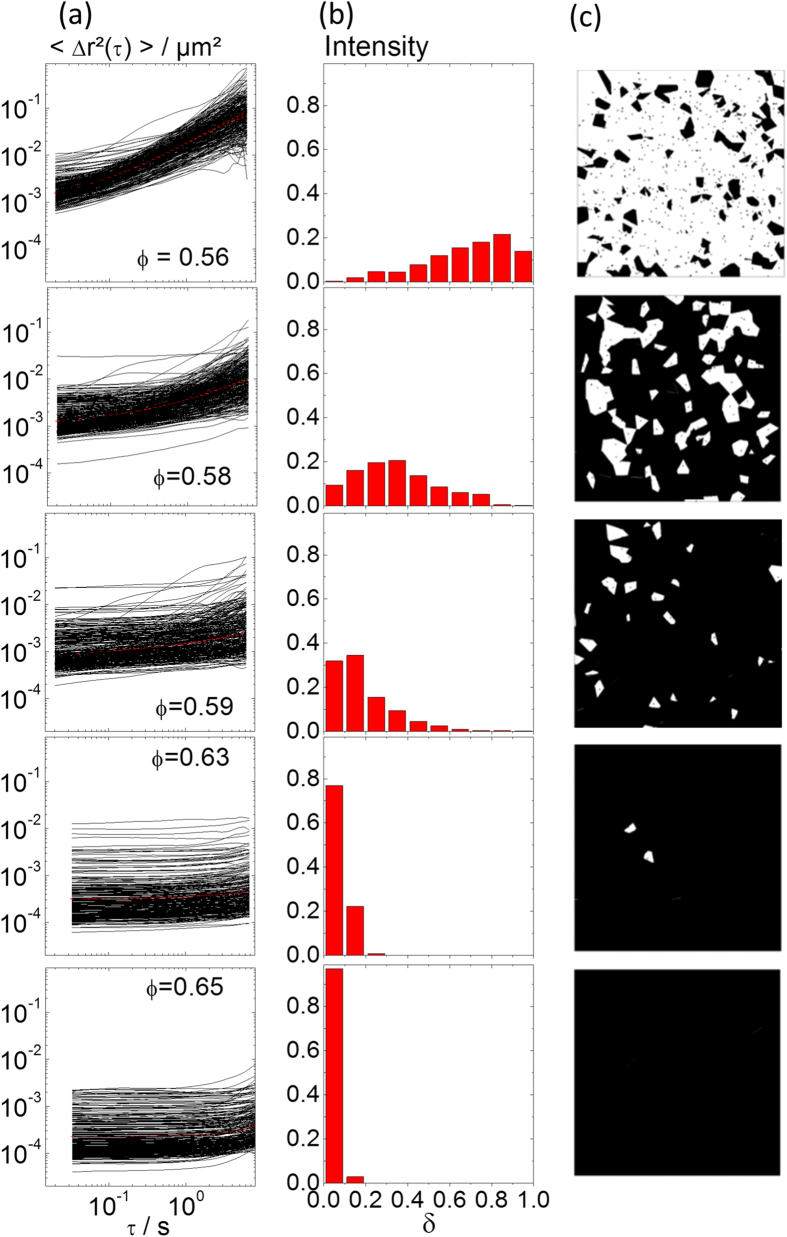
(**a)** MSDs as a function of lag time for the glass-forming dispersion S2 (a = 106 nm) at different volume fractions between ϕ = 0.56 and 0.65. **(b)** Corresponding MSD slope δ distribution measured at lag time τ = 0.5 s. **(c)** Corresponding Voronoi diagram where viscous (white) and elastic (black) regions correspond to δ > 0.5 and δ ≤ 0.5, respectively.

**Figure 7:**
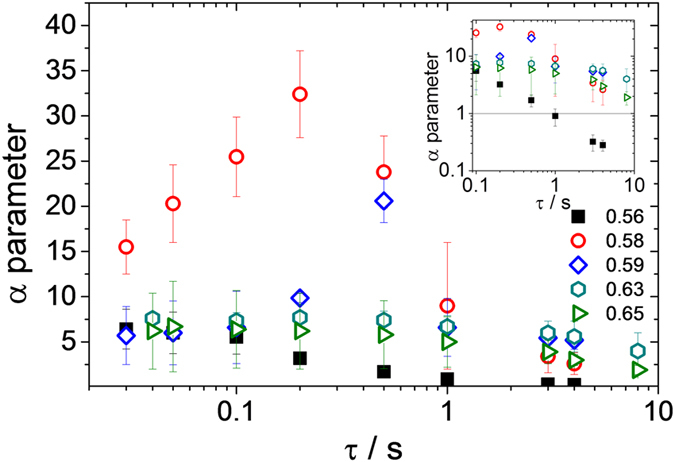
Variation of the non-Gaussian parameter α as a function of lag time τ for the glass-forming dispersion S2 (a = 106 nm) at different volume fractions varying between ϕ = 0.56 and 0.65. Inset: double-logarithmic plot of α versus lag time τ focusing on the static heterogeneities (τ → ∞) observed in the glassy state.

The colloidal glass transition was examined using MPT, and the corresponding data for different particle loadings ϕ between 0.56 and 0.65 are shown in Fig. 6. At low lag times τ, the MSDs were always constant, but for ϕ ≤ ϕ_g,HS_ a large fraction of tracer particles exhibited an increase in MSD, indicating cage escape of individual particles, finally allowing for macroscopic stress relaxation. In the glassy regime (ϕ > ϕ_g,HS_), most probe particles showed time-independent MSDs indicating that the particle configuration was frozen and no stress relaxation was possible, at least on the experimental time scale probed here. This was confirmed by the frequency dependence of macroscopic shear moduli G′ and G″ shown as Supplementary Fig. S3. The transition from a highly heterogeneous system with viscous regions prevailing (ϕ = 0.56) via another heterogeneous state where elastic regions dominate (ϕ = 0.58) and finally to a uniform elastic system (ϕ ≥ 0.63) is revealed in the distribution of MSD slopes δ (taken at τ = 0.5 s) shown in Fig. 6(b) and the Voronoi diagrams in Fig. 6(c). The non-uniform microstructure revealed by the broad distribution of MSDs with different absolute values and time dependence was quantitatively characterized by the parameter α defined in Eq. 4 and the corresponding data are shown in Fig. 7.

In agreement with the pioneering work in this field^11^,^12^, we found a pronounced maximum in α at a characteristic time, especially at the colloidal glass transition (ϕ = 0.58). This phenomenon is called dynamic heterogeneity and was predicted by mode coupling theory (MCT)^46^. The characteristic time at which the maximum occurs is supposed to scale with a^3^ and, based on the order of magnitude smaller particle size used here compared to the early investigations of hard sphere systems using confocal microscopy, this maximum is found at τ values ∼10^3^ times shorter than in the studies mentioned above. In the supercooled state, (ϕ = 0.56) α goes to zero and all tracer particles explore the same environment in the limit τ → ∞ (see insert to Fig. 7). In contrast to the results reported earlier, however, α remains finite and α ≫ 0 even at the longest investigated lag times τ for ϕ > ϕ_g,HS_, indicating that fluctuations in particle concentration are frozen. This phenomenon can be clearly seen here as ∼10^6^ particles are in the field of observation. MCT also predicts the shift of the glass transition to a higher ϕ and the existence of two different glassy states in colloidal systems with weak attractive interactions Ψ_dep_ 47,48,49. The Ψ_dep_ − ϕ phase diagram shows a pronounced curvature of the fluid-glass transition line and at a fixed ϕ > ϕ_g,HS_, a transition from a repulsive glass (particle caging) to a fluid state and then to an attractive glass (particle bonding) is predicted for increasing attraction strength Ψ_dep_ (re-entry glass transition). This was confirmed experimentally via dynamic light-scattering experiments on index-matched suspensions of polymethylmethacrylate^29^ and polystyrene-microgel^50^ particles. The different glassy states were identified by the non-vanishing long-time limit of the dynamic structure factor. The fluid/glass transitions are also observed in macroscopic flow behavior and the re-entry glass transition phenomenon has been used to fluidize technically relevant aqueous polymer dispersions stabilized by short-range repulsion. The low shear viscosity level achieved in an almost monomodal dispersion due to weak depletion attraction was similar to the state-of-the-art freely flowing colloidal dispersions that utilize a broad particle size distribution^41^. Here we used MPT together with bulk rheometry to characterize the different ergodic and non-ergodic states.

**Figure 8:**
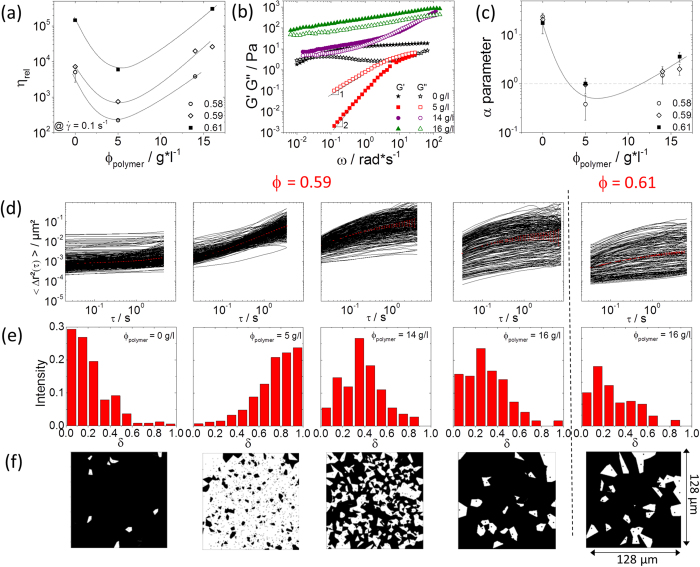
(**a**) Variation of the relative viscosity, normalized by the solvent viscosity, as a function of the addition of non-adsorbing polymer of the dispersion S2 with ϕ = 0.58 (open circles), 0.59 (open diamonds) and 0.61 (closed squares) as obtained from steady shear measurements at low shear rate 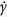 = 0.1 s^−1^. **(b)** Variation of dynamic shear-moduli G′ (closed symbols) and G″ (open symbols) as a function of frequency of a colloidal dispersion (ϕ = 0.59) in the presence of non-absorbing polymer 0 (stars), 5 (squares), 14 (diamonds) and 16 g/l (triangles) as determined from rotational rheometer measurements. **(c)** Variation of α parameter of ϕ = 0.58, 0.59 and 0.61 as a function of the non-adsorbing polymer concentration ϕ_polymer_. **(d)** MSDs variation as a function of lag time from left to right: ϕ = 0.59, 0, 5, 14 and 16 g/l and ϕ = 0.61 with 16 g/l PEO. **(e)** Corresponding MSD slope δ distribution measured at lag time τ = 1 s, and Voronoi diagram **(f)** as obtained from MPT measurements with tracer particles of radius 105 nm.

The re-entry glass transition phenomenon shows up as a pronounced minimum in low shear viscosity upon varying the polymer concentration observed at different fixed values of ϕ > ϕ_g,HS_ (Fig. 8(a)) and the existence of a fluid state is even more evident from the frequency dependence of the linear viscoelastic storage and loss moduli shown in Fig. 8(b). A terminal flow regime with G′ ∼ ω^2^ is clearly seen at an intermediate concentration of non-adsorbing polymer ϕ_polymer_ = 5 g/l. These data also reveal a clear difference between the two non-ergodic states. Without added polymer (repulsive glass), G′ is essentially frequency independent and G′ ≫ G″, the crossover of both moduli at very low frequencies, indicates a still finite relaxation time at this particle loading ϕ ≈ ϕ_g,HS_. This crossover disappears (or is shifted out of the experimentally accessible frequency window) when ϕ is further increased (see Supplementary Fig. S3). Note, this corresponding characteristic relaxation time is approximately five orders of magnitude above the Brownian time scale of an individual particle freely diffusing in water, τ_B_ = a^2^/D ≅ 5*10^−3^s.

The attractive states found at ϕ_polymer_ = 14 g/l and 16 g/l exhibit distinctly different relaxation patterns. G′ and G″ do not crossover and both moduli are higher than in the repulsive glassy state. The strong upturn of both G′ and G″ observed for the ϕ_polymer_ = 14 g/l system at high frequencies indicates the existence of two different relaxation mechanisms. At ϕ_polymer_ = 16 g/l, G′ is larger than G″, but both moduli exhibit a similar weak increase according to the power law G′ ∼ G″ ∼ ω^1/4^, which is valid in a frequency range covering four orders of magnitude. This is a typical signature of a critical gel; in chemically crosslinked networks, this corresponds to the point at which a percolating, sample-spanning network is formed^51^. Finally, it should be noted that stronger depletion forces are required to form attractive arrested states in these colloidal systems stabilized by short range repulsive interactions compared with true hard sphere systems^29^. Further insight is provided by the MPT data shown in Fig. 8(c–e) displaying the individual MSDs, the value of the heterogeneity parameter α, and the distribution of slopes δ (taken at τ = 1 s), respectively. Without added polymer, the MSD traces were almost time-independent at short τ and most δ values were close to zero. The absolute MSD values varied remarkably, however, indicating local variation of particle packing. Some particles exhibited subdiffusive motion at large τ, indicating that the structure was not completely frozen at this ϕ value, marking the onset of the hard sphere glassy regime. At ϕ_polymer_ = 5 g/l, most MSDs were time-dependent with a slope δ > 0.5 demonstrating that the tracers explored a weakly viscoelastic, almost fluid like environment. A characteristic feature of the arrested states observed at ϕ = 0.58 (data not shown), 0.59, and 0.61, and ϕ_polymer_ = 14 g/l and 16 g/l was the initial subdiffusive increase of MSDs and the slow approach of a limiting MSD value. This MSD plateau decreased with increasing ϕ or ϕ_polymer_ and roughly corresponded to the particle radius squared (a^2^ = 1.1*10^−2^ μm^2^). Qualitatively, this seems to be a first experimental confirmation of molecular dynamics simulations also reporting a delayed approach of a limiting MSD value for colloidal systems with weak attractive interactions^52^. In contrast to these simulation results, the limiting MSD value in the attraction-dominated states was larger than for the repulsive glass. Moreover, our relative MSD/a^2^ values ranged between ∼0.1 (repulsive glass) and ∼1 (attractive arrested state), whereas the simulations yielded MSD/a^2^ values ∼0.01. Nevertheless, our experimental data clearly revealed that the particles were trapped in the cage of neighboring particles, but not in the short-range attractive potential well and, according to^52^, the attractive arrested states observed at ϕ_polymer_ = 16 g/l should be termed bonded repulsive glass. The situation is even more complex for the arrested state observed at ϕ = 0.59 and ϕ_polymer_ = 14 g/l: in this case, two distinct populations of particles were observed. A large fraction of tracers seemed to approach a constant finite MSD value at long lag times τ and the slope of the corresponding MSD traces approached zero at long lag times (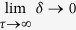), confirming that these particles are trapped in an elastic environment. Other tracers, however, exhibited subdiffusively increasing MSD traces with slopes δ > 0.5 indicating a fluid-like environment. This co-existence of two particle populations is a clear indication of a gel-state. In^52^, such states are termed dense gels, but the MSD simulations do not reveal the existence of two populations with different MSD signatures. The structure of this attractive gel state can be directly imaged using Voronoi triangulation. The white polygons in Fig. 8(f) correspond to tracers in a fluid environment (δ > 0.5), and the black areas correspond to tracers trapped in an aggregated elastic structure (δ ≤ 0.5). The characteristic length scale of this structure was ∼15 μm, corresponding to ∼80 particle diameters.

The degree of structural heterogeneity is reflected in the α values shown in Fig. 8(c) corresponding to the MSD data shown in Fig. 8(d). In both non-ergodic states α ≫ 0 was found irrespective of τ, clearly indicating local particle concentration fluctuations. In contrast, low values (α < 1) were found in the intermediate fluid state, confirming a uniform microstructure where high particle mobility averages out any density fluctuation. The shift in the glass transition volume fraction due to weak attractive interactions predicted by MCT^47^,^48^,^49^,^53^ is reflected in the ϕ-dependence of the α parameter determined at intermediate times at which it should exhibit a maximum. Without added polymer, α > 10 was found at ϕ_g,HS_ = 0.58, when weak attractions were present (ϕ_polymer_ = 5 g/l) such high α-values were observed for ϕ = 0.65, as can be seen in Supplementary Fig. S4.

## Summary and Conclusion

We demonstrated that MPT is a versatile tool for microrheological characterization of highly turbid, concentrated colloidal dispersions. This technically highly relevant class of materials is not accessible with confocal laser scanning microscopy, which has provided fundamental insights into the physics of hard sphere systems using micron-sized model particles in index-matched fluids. We investigated several polymer dispersions, including particles ∼100 nm in radius stabilized by short-range repulsive interactions. Our study covers a broad concentration range from the semi-dilute to the glassy regime. In the fluid state at particle loadings below the colloidal freezing point (ϕ_c_ = 0.495), MPT probes the long-time self-diffusion corresponding to the zero shear viscosity and η_0_ values calculated from MPT data using the generalized Stokes-Einstein equation agree very well with the data from mechanical bulk rheometry. More importantly, MPT allows for direct imaging of micron-sized crystals as well as the characteristic length scales of gel-structures or density fluctuations in glasses using image overlay and Voronoi triangulation. In the fluid-crystalline co-existence region, MPT provides quantitative data for particle concentration in the fluid as well as in the crystalline regions; the latter was confirmed by direct determination of Bragg peaks in light-scattering experiments. The elastic modulus of the crystalline regions was also accessible from the time-independent MSDs of tracers incorporated in the crystals. Dynamic heterogeneities were detected around ϕ_g,HS_ and permanent particle concentration fluctuations for ϕ > ϕ_g,HS_.

Our research especially focused on the effect of weak attractive depletion forces in concentrated colloidal dispersions inferred from non-adsorbing polymer dissolved in the continuous phase. The non-trivial correlation between interaction strength and macroscopic flow behavior could be resolved by combining MPT and classical macroscopic shear rheometry.

At ϕ < ϕ_c_, the transition from a fluid to a fluid-crystalline co-existence regime and finally to a gel-state resulted in a monotonic increase in shear viscosity, especially at low shear rates. This multiple structural transition did not show up in experimental investigations of hard sphere systems at low volume fractions (ϕ < 0.05). In this study, a direct transition from the fluid to the gel state was observed at an attraction strength of a few k_B_T^54^. This direct transition to the gel state is also observed in computer simulations of ideal hard spheres with weak attractive interactions at particle loadings similar to that investigated here^55^. At concentrations corresponding to the fluid-crystalline co-existence of hard spheres, the added polymer affects not only the density, but also the size of the crystal regions. Large highly permeable crystals at an intermediate polymer concentration Ψ_dep_ ∼ k_B_T resulted in a pronounced drop of low shear viscosity, but viscosity increased in the whole investigated shear rate range when dense small crystals formed at a higher attraction strength (Ψ_dep_ ≥ 3.5 k_B_T). The elastic modulus of crystals was determined and the normalized modulus decreased with increasing Ψ_dep_.

The well-known viscosity reduction (i.e., fluidization) of glassy samples due to weak attractive interactions corresponds to a fairly homogeneous fluid state where all tracer particles explore a similar environment. A shift of the maximum of the heterogeneity parameter α indicates a shift of the colloidal glass transition to higher ϕ, as predicted by MCT. The different arrested states observed for ϕ > ϕ_g,HS_ all exhibited strong heterogeneity showing up in α > 0. The repulsive and attractive non-ergodic states also show characteristic differences. For repulsive glass, MSDs are essentially time-independent in the whole investigated τ-range and MSD/a^2^ ≈ 0.1. In contrast, a constant MSD/a^2^ ≈ 1 was slowly approached at large lag times when attractive interactions dominated and, referring to^52^ this is called a bonded repulsive glass. At lower ϕ_polymer_, another arrested state, a so-called dense gel, was observed. In this case, a fraction of particles exhibited monotonically increasing MSDs with δ > 0.5. The characteristic length scale of this gel structure was on the order of 10–20 μm (≈50–100 particle diameters).

In summary, MPT was demonstrated to be a powerful tool for characterizing the microstructure, and dynamic and static heterogeneities in turbid colloidal suspensions, and thus allows for a rational, targeted engineering of the flow, i.e., processing, properties of technically highly important materials. At the same time, this method enables systematic studies of fundamental physical features of soft matter and we retrieved otherwise hardly accessible valuable information about the effect of weak attractive interactions on phase composition, modulus of colloidal crystals, and dynamic and static heterogeneities in different arrested and intermittent fluid states.

## Methods

All investigated dispersions included colloidal particles with short-range repulsive interactions dispersed in water. The crystallizing dispersion S1 comprised poly(styrene-butyl acrylate) P(S/BA) particles with 2%wt relative to the total monomer concentration of acrylic acid as a surface functional co-monomer. The particle radius was 130 nm and the polydispersity below 3%. This dispersion was provided by BASF SE. S2 was a glass-forming technical anionic, aliphatic polyester-polyurethane dispersion provided by Covestro. The mean particle radius was 106 nm. Two additional technical dispersions, poly(styrene-acrylate) and an anionic poly(carbonate-urethane) dispersion with a = 54 nm and a = 15 nm, respectively, were included for the comparison between bulk viscosity η_0_ and microviscosity η_MPT_ at various volume fractions.

Different chemical modifications on the surface provided repulsive interactions to stabilize the dispersion. The determined volume fraction ϕ was calculated via the solid content x_particle_ and the measured density of the dispersion ρ_dispersion_ with ϕ_core_ = (x_particle_ * ρ_dispersion_)/ρ_particle_. The effective volume fraction ϕ_eff_ was then obtained from the divergence of the zero shear viscosity determined for a series of semi-dilute and concentrated dispersions. Table 2 summarizes the characteristic sample specifications.

Non-adsorbing PEO (Merck, Germany) with a molecular weight of 20 kDa (according to the supplier) was added to the samples in different concentrations to introduce weak attractive depletion interactions. The corresponding radius of gyration R_g_ in water, calculated according^56^, was 6.3 nm. The overlap concentration c* of this molecular weight is 88 g/l according to^57^.

According to^58^, we calculated the depletion potential between two particles in contact as


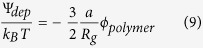


where ϕ_polymer_ is the volume concentration of the polymer in the liquid phase, estimated as


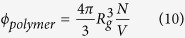


where N denotes the total number of polymer molecules in solution and V is the total volume of the liquid phase. Depending on the polymer concentration and particle size, the depletion interaction varied between 1 and 7 k_B_T. Table 3 shows all of the data.

Concentrated dispersions including the polymer dissolved in the continuous phase were prepared via a dialysis procedure. A dialysis membrane (Carl Roth, Germany) with a molecular weight cut-off of 4–6 kDa was filled with the dispersion and immersed in a dialysis bath filled with an aqueous solution of PEO, M_w_ = 35 kDa, at a concentration of 15 %wt. The high osmotic pressure of the PEO solution concentrates the dispersion to the desired ϕ. The pH of the solution was adjusted to 7 by adding 1 M NaOH.

### Macroscopic Rheological Characterization

Static shear measurements were performed using a strain-controlled ARES Rheometer (TA Instruments) with a Couette geometry (34 mm outer diameter, 1 mm gap width). The samples were covered with low viscosity paraffin oil to prevent evaporation. The shear rate was varied between 0.1 and 500 s^−1^. Oscillatory shear experiments in the linear viscoelastic regime were performed on a stress-controlled Physica MCR501 (Anton Paar) using cone-plate geometries of different diameters (25 mm/2° and 50 mm/1°) depending on the stiffness of the sample. The frequency range was between 0.01 and 150 rad/s. All measurements were accomplished at a temperature of 20 °C and every measurement was preceded by a waiting time of 5 minutes to relax the residual stress produced by the filling process.

### Passive Microrheology

MPT was performed to investigate the micro-structure and local dynamics of the colloidal dispersion. For this, 0.5 μL of a dispersion containing 1%wt. fluorescent tracer particles (a = 105 nm, dragon green) from Bang Laboratories (USA) was added to 100 μL of the colloidal dispersion, which was then vortexed and homogenized for 10 min in an ultrasonic bath. A 20 μL aliquot of this mixture was then injected into a small probing chamber that we constructed^59^ and sealed with UV-curing glue. The MPT experiments were performed with an inverted fluorescence microscope (Zeiss Axio Observer D1) equipped with a Fluar 100x, N.A. 1.3, oil-immersion lens combined with a 1x optovar magnification charger. An LED light source (Colibri, Zeiss) was connected to the microscope, providing the required wavelength of 475 nm. One thousand consecutive images of the Brownian motion of the tracer beads were recorded onto a personal computer with an sCMOS camera Zyla X (Andor Technologies: 2048 × 2048 square pixels, up to 50 frames/s in global shutter mode), and, depending on the tracer particle velocity, a frame rate between 30 and 50 fps was selected. After image processing (IPS Visiometrics), the displacement of particle centers was monitored with self-written MatLab code^23^, using the widely used Crocker and Grier tracking algorithm^60^ in a 128 × 128 μm field of view. An overlay of all 1000 images gave additional information about the crystal size and shape in the case of crystalline samples.

### Light Diffraction Experiments

Bragg-reflection experiments were performed in Prof. PalberG’s laboratory (Johannes Guttenberg Universität Mainz). Samples were filled in rectangular quartz cells with a cross section of 10 × 10 mm^2^, as used in ref. 61. Crystallization was monitored by static light-scattering using a fiber spectrometer (Avantes AvaSpec-2048 with Avantes FCR as glassfiber), wavelength range between 280–841 nm, and an AvaLight (Avantes) light source provided the required wavelength between 215–1500 nm. Details of the setup are described in ref. 62. The obtained data were processed with the spectrometer software AvaSoft7.3.

## Additional Information

**How to cite this article**: Weis, C. *et al.* Microstructure, local dynamics, and flow behavior of colloidal suspensions with weak attractive interactions. *Sci. Rep.* **6**, 33498; doi: 10.1038/srep33498 (2016).

## Supplementary Material

Supplementary Information

## Figures and Tables

**Table 1:** Characteristic data for sample S1 at different particle volume fractions ϕ and polymer concentrations ϕ_polymer_.

ϕ	ϕ_polymer_	η_0_	η_fluid_	ϕ_solid_	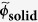	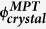	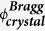
	g/l	mPas	mPas				
0.45	15	53.4 ± 6	3.81 ± 1.56	0.51 ± 0.03	0.44 ± 0.03	0.61 ± 0.02	
0.52	3	48 ± 15	28 ± 7	0.71 ± 0.03	0.2 ± 0.05	0.54 ± 0.02	
	10	1560 ± 150	9 ± 2	0.56 ± 0.07	0.54 ± 0.01	0.64 ± 0.04	0.65 ± 0.008
0.54	3	72 ± 16	42 ± 7	0.73 ± 0.04	0.14 ± 0.01	0.56 ± 0.02	0.584 ± 0.007
	10	5100 ± 300	9 ± 2	0.6 ± 0.03	0.56 ± 0.01	0.64 ± 0.04	0.646 ± 0.009

Zero-shear viscosity η_0_ is determined from bulk rheometry, η_fluid_, ϕ_solid_, and 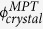 are deduced from MPT; 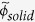 is calculated from Eq. 5 using η_0_ and η_fluid_; and 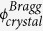 is obtained from light diffraction experiments.

**Table 2:** Chemical composition, surface modification, particle radius a, effective volume fraction ϕ of the investigated samples.

Name	Chemical composition	Stabilization	a in nm	ϕ
S1	P (S/BA)	Acrylic acid	130	1.076ϕ_core_
S2	PU	Carboxyl acid ester	106	1.179ϕ_core_
S3	P (S/A)	Acrylic acid	54	1.061ϕ_core_
S4	P (S/BA)	Acrylic acid	90 & 125	1.07ϕ_core_
S5	PU	Carboxyl acid ester	15	1.44ϕ_core_

**Table 3:** Investigated polymer concentrations, polymer-to-particle size ratio ξ = R_g_/a, and resulting depletion interaction energy Ψ_dep_/k_B_T for the crystallizing sample S1 and the glass-forming dispersion S2.

Dispersion	ϕ_polymer_ in g/l	ξ	Ψ_dep_/k_B_T
S1	3	0.049	1.05
	5		1.76
	10		3.52
	15		5.26
	20		7.03
S2	5	0.059	1.43
	14		4.02
	16		4.6
