# Supplementary material for: Microstructure, local dynamics, and flow behavior of colloidal suspensions with weak attractive interactions
Source: Sci Rep. 2016 Sep 22;6:33498. doi: 10.1038/srep33498 (PMC5031965; doi:10.1038/srep33498)
Supplement: Supplementary Information [file srep33498-s1.pdf]

# Microstructure, local dynamics, and flow behavior of colloidal suspensions with weak attractive interactions

**Clara Weis [1*], Claude Oelschlaeger [1], Dick Dijkstra [2], Meik Ranft [3] and Norbert Willenbacher [1]**

[1] Karlsruhe Institute for Technology (KIT), Institute for Mechanical Process Engineering and Mechanics, Applied Mechanics, Karlsruhe, 76131, Germany

[2] Covestro Germany, Leverkusen, 51365, Germany

[3] BASF SE, Ludwigshafen, 67056, Germany

[*] clara.weis@kit.edu

**Supplementary Information**

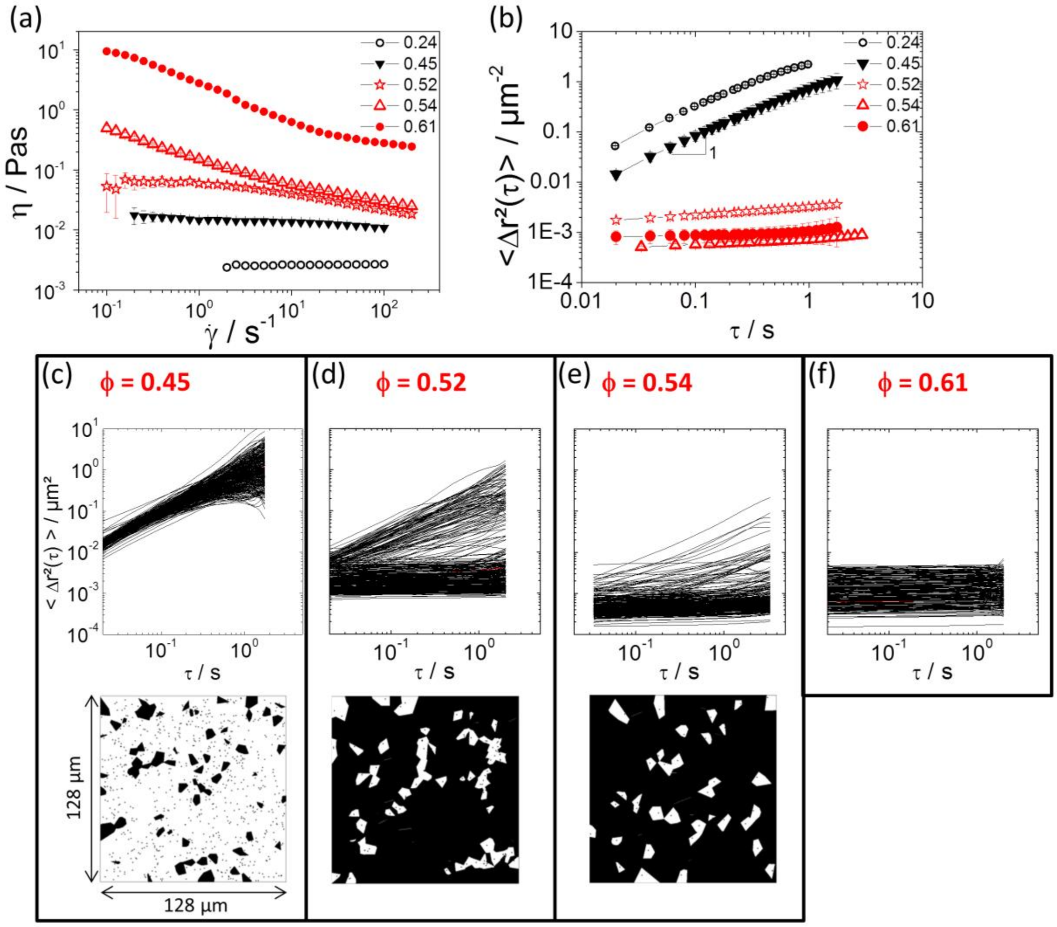


**Figure S1. (a)** Viscosity versus shear rate $\dot{\gamma}$ of dispersion S1 for volume fractions $\phi$ between 0.24 and 0.61. **(b)** Mean MSD versus lag time $\tau$ for the volume fractions shown in (a). **(c)-(f)** MSDs traces and corresponding Voronoi diagrams where viscous (white) and elastic (black) regions correspond to $\delta > 0.5$ and $\delta \leq 0.5$, respectively, for volume fraction 0.45 (c), 0.52 (d), 0.54 (e), and 0.61 (f).

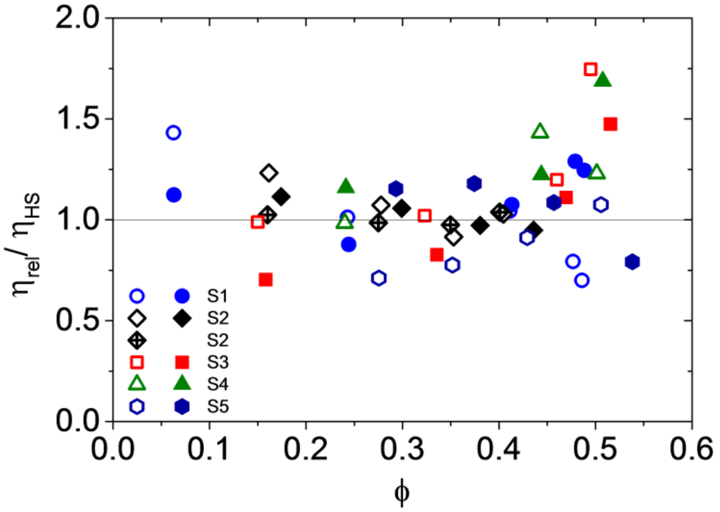


**Figure S2.** Deviation of experimental data from the Maron & Pierce model (Eq.5) for the bulk- and micro-viscosity data shown in Figure 1.

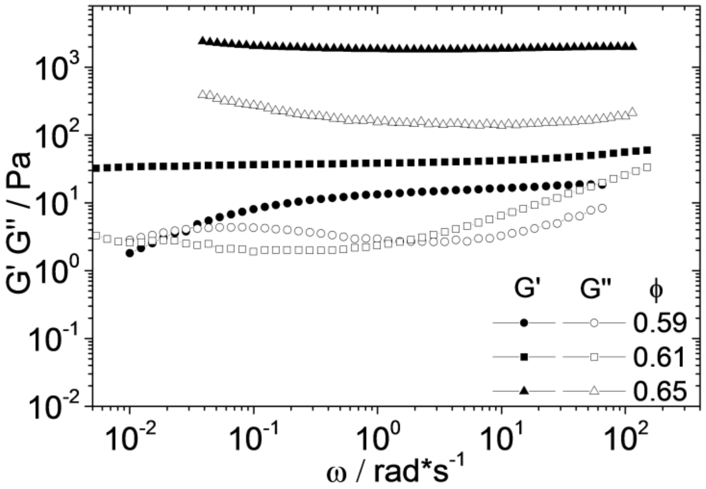


**Figure S3.** Macroscopic shear moduli G' (closed symbols) and G" (open symbols) as a function of frequency $\omega$ for dispersion S2 at $\phi$ = 0.59 (circles), 0.61 (squares), and 0.65 (triangles).

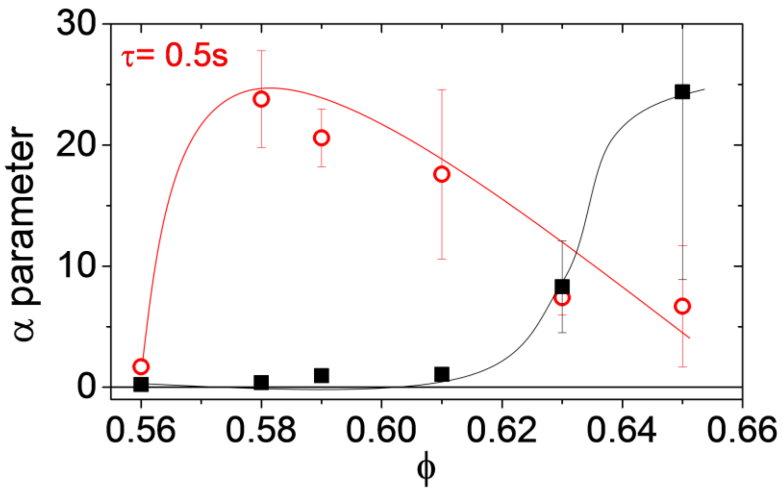


**Figure S4.** Non-Gaussian parameter $\alpha$ as a function of volume fraction $\phi$ for dispersion S2 with 5 g/l added PEO (black squares) and without added polymer (red circles) determined at lag time $\tau$ = 0.5 s. The lines serve as a guide to the eyes.
